# First person – Anabel Byars

**DOI:** 10.1242/bio.062124

**Published:** 2025-07-22

## Abstract

First Person is a series of interviews with the first authors of a selection of papers published in Biology Open, helping researchers promote themselves alongside their papers. Anabel Byars is first author on ‘ Unpredictable disturbance and its effects on activity behavior and lifespan in *Drosophila melanogaster*’, published in BiO. Anabel conducted the research described in this article while an undergraduate research assistant in Dr Nicole Riddle's lab at the Department of Biology, The University of Alabama at Birmingham (UAB), USA. She is now an incoming PhD student in graduate biomedical sciences (neuroscience theme) at UAB and will start her PhD program in July 2025, at which point she will complete several lab rotations before choosing a new lab and PI in the Department of Neurobiology at the same institution. Anabel has a range of research interests, including Alzheimer's disease, addiction, memory, nonpathological aging, and neuroplasticity.


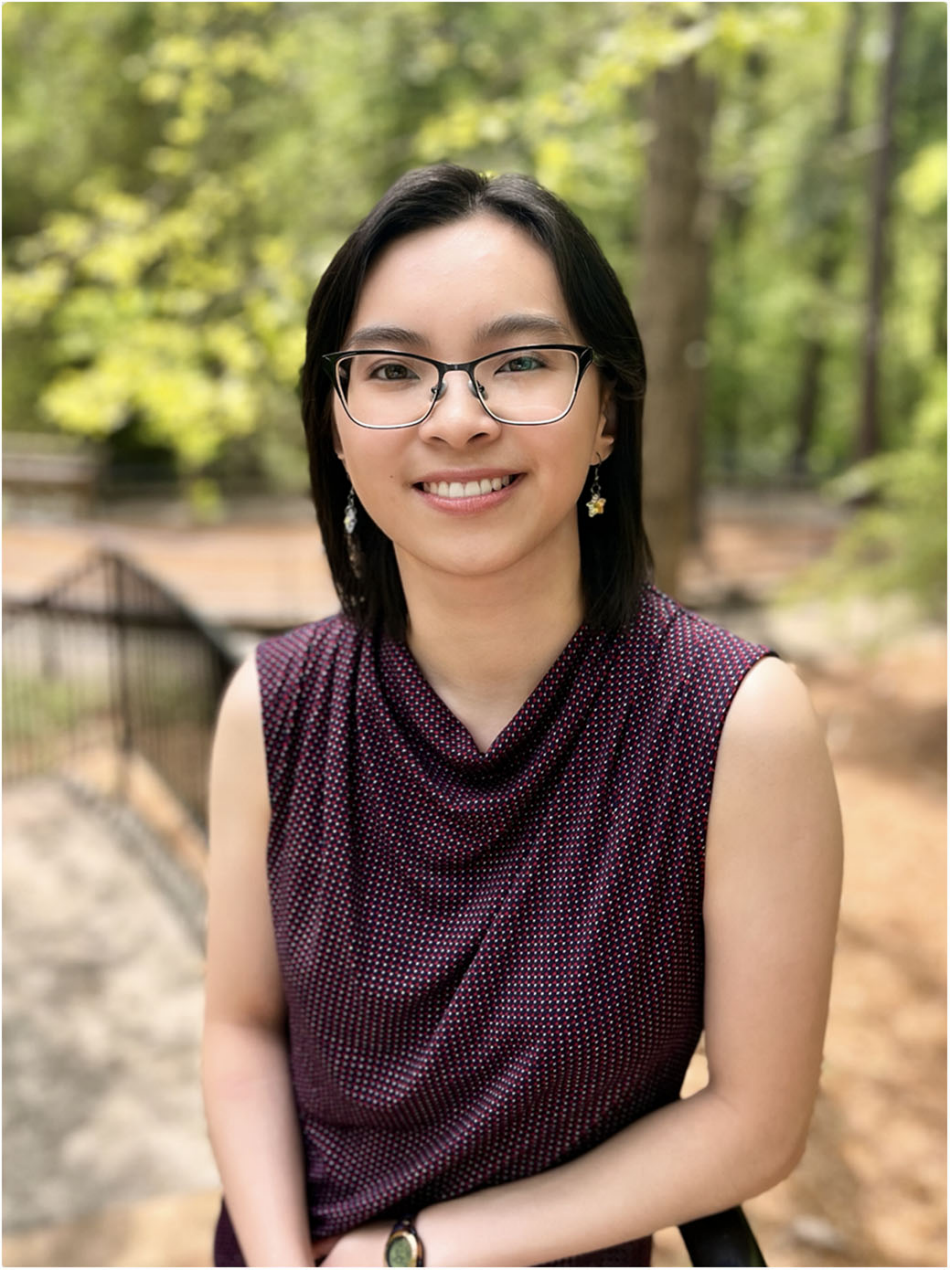


**Anabel Byars**

**Describe your scientific journey and your current research focus**

My journey in science started when I enrolled at UAB and entered the undergraduate neuroscience program. At the start, I was a dedicated pre-med student, meticulously completing shadowing and volunteer work, while trying to maintain a stellar GPA. I joined a research lab during the summer after my freshman year, determined to further boost my resumé. Instead, the move altered my career trajectory. I spent my remaining fall, spring and summer semesters with fruit flies. Under the guidance of my lab mentors, I worked on several projects, including investigating the effects of unpredictable mechanical disturbance on activity and lifespan and determining whether exercise provides health benefits to a *Drosophila* cancer model. Doing hands-on science in the lab reinforced and added to what I was learning in the classroom. Plus, I found my adaptability, determination and creativity tested. In my junior year, I officially decided to pursue a PhD in neuroscience. I am excited to continue my studies at UAB, where I will explore my interests in Alzheimer's disease, addiction, memory, nonpathological aging, and neuroplasticity.

“I joke that the reason I decided to become a scientist was because my mom told me to, and I always do what my mom tells me to do. She hasn't steered me wrong yet!”

**Who or what inspired you to become a scientist?**

I joke that the reason I decided to become a scientist was because my mom told me to, and I always do what my mom tells me to do. She hasn't steered me wrong yet! In all seriousness, I give credit to both of my parents, who prioritised creativity and exploration in my education – skills that ultimately fostered my interest in science and continue to serve me well in the lab.

**How would you explain the main finding of your paper?**

Human disturbances, such as habitat destruction and recreation, have a range of effects on wild animals. Some human activities are harmful while others have little impact. We explored this complexity by mimicking unpredictable human disturbance in a lab setting. Fruit flies were exposed to random, physical disruptions over two different treatment lengths. Four genetically distinct groups of fruit flies and both sexes were used. Not all the flies responded the same to disturbance treatments. Individual differences in genetic background and sex, significantly influenced how the flies responded. Our finding demonstrates that an animal's ability to adapt to environmental stressors, like human disturbance, depends greatly on its unique traits. Overall, predicting the effect of human disturbance on wild animals is complex.

**What are the potential implications of this finding for your field of research?**

This work serves as an example of how lab-based research can be utilised to inform human disturbance studies conducted in wild populations. In the lab, we can test specific conditions like different intensities or types of disturbance, various health measures, and more. Taking a lab-based approach can provide a deeper understanding of how disturbance affects animals and may provide strategies for reducing potentially harmful effects.

**Figure:**
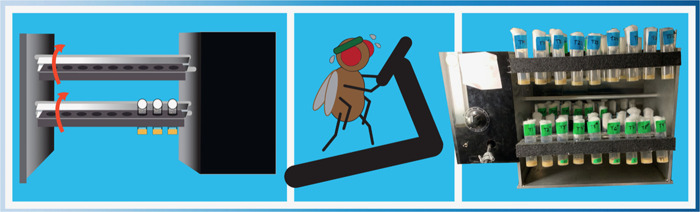
The TreadWheel device was used to mechanically disturb the fruit flies.

**Which part of this research project was the most rewarding?**

The moment when I successfully ran my data through RStudio and saw all that hard work come together in a graph.

**What do you enjoy most about being an early-career researcher?**

As an early-career researcher, I have so many opportunities. I enjoy the freedom of choosing which research topics to explore and learning from experts in my field.

**What piece of advice would you give to the next generation of researchers?**

Accept that being wrong is an essential part of discovery and in no way defines your abilities.

**What's next for you?**

This July I am headed to Dauphin Island for my first course in UAB's graduate biomedical sciences program – neuroscience theme. I look forward to kicking off my PhD with some neuroscience fundamentals on the beach.
